# Commentary: A Neurodynamic Perspective on Musical Enjoyment: The Role of Emotional Granularity

**DOI:** 10.3389/fpsyg.2018.00457

**Published:** 2018-04-05

**Authors:** Charlotte L. Doyle

**Affiliations:** Psychology Faculty, Sarah Lawrence College, Bronxville, NY, United States

**Keywords:** music, cognition, emotion and music, musical enjoyment, phenomenology, neurodynamics

## Introduction

To facilitate discovery of the neurodynamics underlying a listener's emotional response to music, Barrett and Schulkin (2017) take up the question of why we enjoy sad music. They find the answer in the high emotional granularity evoked by the complexity of the sensory-motor response as listeners both perceive “sad” motion and become rhythmically entrained. This commentary proposes two additional phenomenological factors in the enjoyment of sad music, providing new avenues for studying the neurodynamics of listening.

## “Sad” music more than sad

Taruffi and Koelsch (2014) found that even self-identified sad music may evoke a variety of feelings: nostalgia, peacefulness, sadness, tenderness, wonder, and/or transcendence. That research also suggested that these feelings may be related to the capacity for empathy—feeling with *another*, implying that music itself may be a virtual Other. Molnar-Szakacs and Overy (2006) propose a similar hypothesis on the neurodynamic level: music engages the mirror neuron systems related to both motion and emotion—a proposal to be tested empirically. Vuoskoski and Eerola (2017) added evidence that empathy is engaged in listening to sad music; they also reported research which showed that the relation between felt sadness and musical enjoyment was primarily mediated by the feeling of being moved.

The ratings in the studies were overall ratings. But the experience of music changes over time. Musicians have referred to musical works as journeys and, with multiple instruments, as conversations (Berliner, 1997). As it unfolds, the musical structure may bring a cascade of feeling—those just mentioned but also anticipation, suspense, surprise, awe, fulfillment, resolution, and/or completion (Vuust and Frith, 2008). Given the development of moment-to-moment brain recording (e.g., Garrett et al., 2013) and the nervous system differentiation of empathetic emotion (see for example, Rymarczyk et al., 2018), music's temporal structure suggests research exploring neurodynamic and facial muscle changes over time while listening to sad music, looking for biological signatures of various feelings, such as surprise and sadness.

## Listeners in a world of sound

Phenomenologist Schütz (1945) pointed out that human experience can be understood as taking place in multiple realities, e.g., the everyday world, the world of dreams, the theoretical world, the child's play world. Musicians have referred to their music-making as taking place in a sound world (Sessions, 1952; Berliner, 1997). As one musician put it, “From the very moment the performance begins, you plunge into that world of sounds. It becomes your world instantly and your whole consciousness changes” (Berliner, 1997, p. 11).

Each province of meaning involves a unique pattern of features including: the dominant form of action, the sense of self, the experience of time, sociality, and epoché (what is taken for granted). The everyday world is what people take for granted as reality. It is the world of purposive action involving executive functions, acting bodily with intention in and on the world to fulfill goals; the self is the historical self with a remembered past and an anticipated future and includes judgments of self-esteem and self-efficacy. The everyday world is social with interactions with others that can alter sense of self. The everyday sense of time (inner time) is often connected to outer time—the time of clocks Schütz (1945).

When listeners immerse themselves in a musical sound world, these features change. They are in a world shared with performers, perhaps audience, and even the composer across time. What is shared is the experience of the musical flux (Schütz, 1951). The listener is not acting in and on the everyday world to achieve goals (Scherer and Zentner, 2008). There is perceived movement which mirrors the structure of emotion (Sievers et al., 2013); listeners sometimes move bodily to the music (tapping, “conducting,” dancing, etc.), but the movement is part of participating in the sound world, not acting in and on the everyday world. The music creates its own experience of time. The experienced past is the music that flowed before, the future is the anticipation of what is coming next (Schütz, 1951). The music world in sad music is safe (Smuts, 2007); the everyday self with its fears and sorrows has disappeared or moved to the fringes of consciousness. Since musical immersion takes place in a sound world, even when sadness is part of the emotional cascade, it does not arise from the cognitive appraisal of events in the everyday world (Levinson, 1997). Immersive listening has no implications for the everyday self, has no effects on self-esteem, or self-efficacy, brings no need to deal with an everyday sad situation. Rather it is a trip into another world of experience with its own properties, a temporary yet moving respite from the “real” world (Taruffi and Koelsch, 2014)–a major feature underlying its enjoyment. This analysis suggests research comparing the neurodynamics of immersive listening (sound world) with listening in order to analyze the music (the theoretical world which engages executive functions). The multiple reality description would hypothesize that listening to analyze would show greater activation of the lateral prefrontal cortex, an area associated with executive functions (Barbas, 2016), than immersive listening.

## Conclusions

If neurodynamic complexity characterizes the brain of a person immersed in “sad music,” it represents the physiological parallel to the complexity of the psychological experience, a rich vein for future research. Sadness is likely to be only one in the cascade of a listener's intricate, changing feelings over time. Listeners feel moved by the experience. Most centrally, musical immersion brings emotional experience that is qualitatively different, one that is safe, free from challenges of the everyday life, from continual appraisal of its implications for well-being, from intended action to meet demands and goals, and from a self at stake. Thus, we can enjoy the musical flux of “sad” music as we inhabit a socially shared, temporally patterned, emotionally evocative, moving, changing world of sound.

## Author contributions

CD is the sole author and is responsible for the content.

### Conflict of interest statement

The author declares that the research was conducted in the absence of any commercial or financial relationships that could be construed as a potential conflict of interest.
